# Co-Solvents as Stabilizing Agents during Heterologous Overexpression in *Escherichia coli* – Application to Chlamydial Penicillin-Binding Protein 6

**DOI:** 10.1371/journal.pone.0122110

**Published:** 2015-04-07

**Authors:** Christian Otten, Stefania De Benedetti, Ahmed Gaballah, Henrike Bühl, Anna Klöckner, Jarryd Brauner, Hans-Georg Sahl, Beate Henrichfreise

**Affiliations:** Institute for Pharmaceutical Microbiology, University of Bonn, Bonn, Germany; University of Lausanne, SWITZERLAND

## Abstract

Heterologous overexpression of foreign proteins in *Escherichia coli* often leads to insoluble aggregates of misfolded inactive proteins, so-called inclusion bodies. To solve this problem use of chaperones or *in vitro* refolding procedures are the means of choice. These methods are time consuming and cost intensive, due to additional purification steps to get rid of the chaperons or the process of refolding itself. We describe an easy to use lab-scale method to avoid formation of inclusion bodies. The method systematically combines use of co-solvents, usually applied for *in vitro* stabilization of biologicals in biopharmaceutical formulation, and periplasmic expression and can be completed in one week using standard equipment in any life science laboratory. Demonstrating the unique power of our method, we overproduced and purified for the first time an active chlamydial penicillin-binding protein, demonstrated its function as penicillin sensitive DD-carboxypeptidase and took a major leap towards understanding the “chlamydial anomaly.”

## Introduction

The standard procedure to obtain a particular protein comprises cloning of its gene into an appropriate vector, transformation into a chosen host and, after induction of expression, purification. High expression levels frequently lead to the formation of inclusion bodies harboring solely the protein of interest but only small amounts in its active form [1,2]. This is in particular true for proteins which are evolutionary far afield from the expression host and thus adapted to a physico-chemically different environment.

Reasons for the formation of inclusion bodies are closely related to the thermodynamical stability of the protein [3] leading us to exploit methods usually used to stabilize proteins *in vitro*. In the process of biopharmaceutical formulation, a whole bunch of different substances is known and in use to stabilize biologicals for human administration [4]. These so called co-solvents belong to different chemical classes, including sugars, polyols, methylamins or amino acids. Dialysis experiments revealed that the protein stabilizing ability of the co-solvent is facilitated by weak interaction with the protein in the presence of a solvent, which is mostly water or an aqueous solution [5]. These co-colvent:protein:solvent interactions lead to the exclusion of the co-solvent from the surface of the protein [5]. In the field of pharmacy the technical term is “preferential exclusion”. The underlying mechanisms of this principle are discussed later and are described in detail in the review of Timasheff [5]. Resources on substances commonly used as co-solvents in biopharmaceutical formulation are given in [6].

Heterologous overexpression in the periplasm offers considerable advantages compared to other methods. Primarily, translocation over the cytoplasmic membrane customizes the protein of interest to stabilizing agents that are exogenously administrated and gain access to the periplasm, but are usually not able to enter the cytoplasm in high amounts. Furthermore, the periplasm contains less contaminating proteins and possesses a smaller and different set of proteases making purification much easier [7].

Here, we describe a novel method for the heterologous production of recombinant proteins in *E*. *coli* exploiting the stabilizing effects of co-solvents routinely during overexpression in the periplasm, i.e. already *in vivo*. The optimal substance for the protein of interest is empirically determined in a co-solvent screen as the stabilizing effect depends on specific co-solvent: protein interactions. Notably, the co-solvents are used under stress free conditions to overcome negative stimuli caused by changes in osmotic pressure, pH or temperature. Easy accessibility of the periplasm for various substances allows us to use the periplasmic cell compartment as a kind of biopharmaceutical test vial that provides the aqueous part of the tripartite system of co-solvent, protein and solvent.

We used our co-solvent assisted method to purify for the first time a chlamydial penicillin-binding protein (PBP) in its active form. Chlamydiae are an evolutionary separated group of obligate intracellular bacteria which replicate inside of a host derived vacuole (inclusion) to escape clearance by the immune system. PBPs serve as the putative targets of penicillin in chlamydiae [8] and are thus key proteins to elucidate the chlamydial anomaly; a paradox that describes the susceptibility of *Chlamydiaceae* towards penicillin in the absence of a cell wall envelope [9]. To gain mechanistic insights into the chlamydial anomaly we overexpressed PBP6 from the human pathogen *Chlamydia pneumoniae* in the presence of betaine and identified the purified protein as functional DD-carboxypeptidase cutting D-Ala residues from cell wall building blocks.

## Materials and Methods

### Cloning into periplasmic expression vector

**Fig 1:**
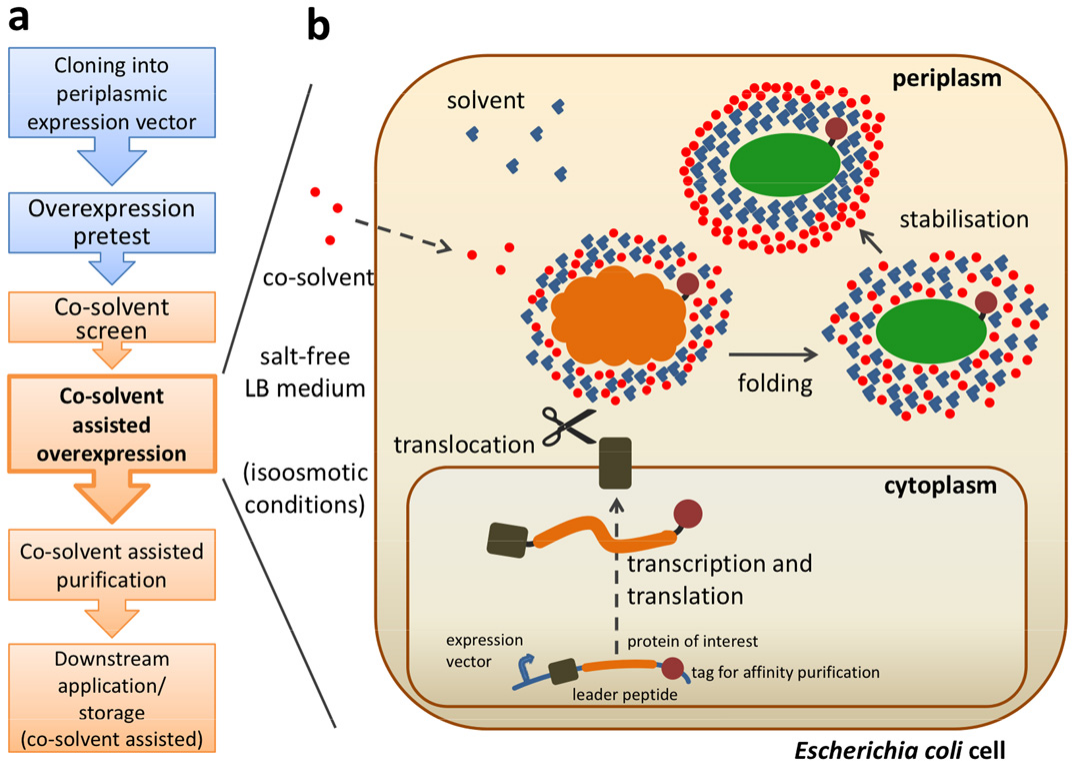
Systematic use of co-solvents in heterologous overexpression and purification of proteins. (a) Workflow for the co-solvent assisted overproduction and purification method and (b) cartoon on the step of co-solvent assisted overexpression in the *E*. *coli* periplasm illustrating the mode of action of co-solvents.

**Fig 2:**
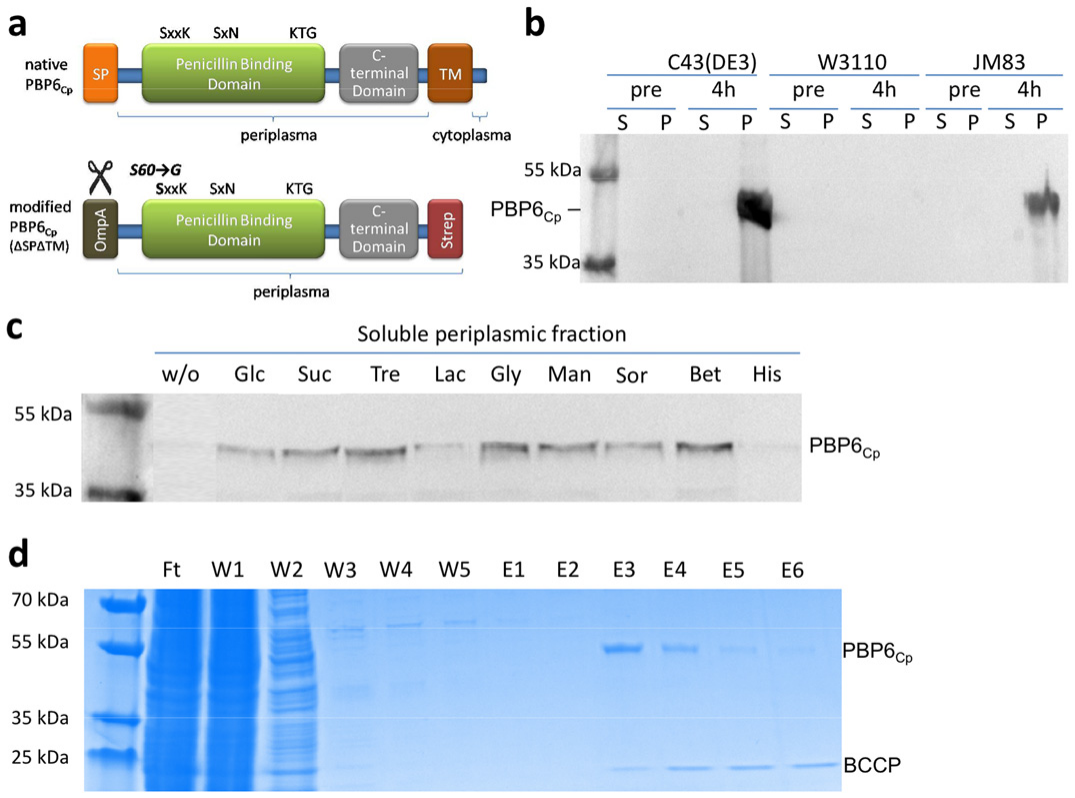
Application of the co-solvent assisted method to PBP6_Cp._ (a) Structure of PBP6_Cp_. Domains, motifs, signal peptides (SP), transmembrane domains (TM), leader peptide sequence for transportation into the periplasm (OmpA) and chromatography affinity tag (Strep) are depicted (SP and TM were predicted by Signal P and TMHMM [30,31]. The modified PBP6_Cp_ (42.95 kDa) which lacks the native SP and TM domain was overproduced, purified and tested for DD-carboxypeptidase activity. (b) Results from overexpression pretest (western blot), (c) co-solvent screen western blot), and (d) betaine-assisted purification (Coomassie stain) of PBP6_Cp_. The optimal expression conditions (*E*. *coli* C43(DE3), 4h of induction at 25°C) and co-solvent (betaine) determined in the overexpression pretest and co-solvent screen, respectively, were used in an up-scaled culture to overproduce soluble PBP6_Cp_ for the first time. Pre: pre induction, S: soluble fraction, P: pellet fraction, w/o: without the addition of co-solvent, Glc: glucose, Suc: sucrose, Tre: trehalose, Lac: lactose, Gly: glycerol, Man: mannitol, Sor: sorbitol, Bet: betaine, His: histidine. BCCP (21.5kDa): biotin carboxyl carrier protein from *E*. *coli*, a common contamination of strep-tagged proteins (biotinylated protein binding to strep-tactin which can be removed by complexation with avidin from hen egg white [12]).

The PBP6_Cp_ encoding gene *dacF* from *Chlamydia pneumoniae* strain GiD [10] was amplified by PCR using primer PBP6ΔSPΔTM_for_Cp and PBP6ΔSPΔTM_rev_Cp (Table A in S1 File) and cloned into pASK-IBA2C (IBA, Germany) resulting in vector pASK-IBA2C_PBP6Cp (Table A in S1 File) that allows for periplasmic expression of PBP6. The sequences coding for the intrinsic N-terminal signal peptide and the C-terminal transmembrane domain was removed and the gene was N-terminally fused to leader peptide OmpA and a Strep tag was added to the C-terminus (Figs 1b and 2a).

### Site-directed mutagenesis

S60 in PBP6_Cp_ was changed to G using the QuikChange Lighting Site-Directed Mutagenesis Kit (Agilent Technologies, Germany). The sense and antisense primers PBP6ΔSPΔTM_S60G_for_Cp and PBP6ΔSPΔTM_S60G_rev_Cp (Table A in S1 File) were used according to the manufacturers’ instructions. Correct base changes were confirmed by sequencing.

### Small-scale overexpression pretest

Initially, expression pretests are used for systematic identification of conditions that facilitates the production and yield the highest amounts of (aggregated) protein. Variation of the *E*. *coli* expression strain (*E*. *coli* K-12 subsp. W3110, *E*. *coli* BL21 C43, and *E*. *coli* JM83 (Table B in S1 File)), the growth temperature (25 and 30°C) and the time of induction (4h, over night) was used to affect protein overexpression.

Small-scale overexpression pretests were performed using 30 ml of standard LB medium containing the appropriate antibiotic. Induction was carried out at an OD_600_ of 1.2 with 200 ng/ ml of anhydrotetracycline. Samples were taken after 4h and overnight for all tested strains and temperatures. The periplasmic fraction was isolated using the method of Withold *et al*. with slight modifications [11]. The cell pellet was resuspended in 100 mM Tris/HCl pH 8, 500 mM sucrose, 1 mM EDTA and 1 mg/ml lysozyme. The suspension was incubated on ice for at least 30 minutes. To reduce viscosity benzonase in a concentration of 20 U/ml was added and incubated for additional 15 minutes on ice. The periplasmic fraction was isolated from the spheroplasts by centrifugation. Periplasmic proteins were separated by SDS-PAGE and the overexpressed protein was detected by Western Blot using antibodies that are specific for the fused affinity tag [12].

### Small-scale co-solvent screen

Following the identification of the optimal combination of expression strain, temperature and and time using the pretests described above, our method includes a co-solvent screen as the stabilizing effects depend on specific interactions between co-solvent and protein. The optimal co-solvent was determined empirically for PBP6_Cp_ as decribed below.

A single colony of freshly transformed plates was picked to inoculate 30 ml of LB-medium containing the desired antibiotic. The preculture was incubated over night under shaking at the permissive temperature. For the screen, cultures with 30 ml of no salt LB medium (lacking the standard amount of 10 g/L sodium chloride), each containing the desired antibiotic and one of the nine co-solvents listed in Table 1, were inoculated with 1% (v/v) of the preculture. To prevent osmotic stress the amount of co-solvent was adjusted to an osmotic pressure elicited by 10 g/L of sodium chloride; i.e. 171 mM sodium chloride which are equivalent to 342 osm/L of dissociated Na^+^ and Cl^-^ ions were replaced by 342 mM of non dissociating co-solvents (equivalent to 342 osm/L). To avoid toxic effects, amino acids were added in concentrations of 50 mM and supplemented with 292 mM sucrose. For heat labile co-solvents such as sugars and methylamines a concentrated no salt-LB medium was autoclaved and subsequently supplemented with a sterile-filtered, water-based co-solvent solution.

**Table 1:** Most common co-solvents in biopharmaceutical formulation included in the co-solvent screen described in this study.

	Applied concentration in the growth medium	Effect [6]
**Sugars—monosaccharides**		
Glucose	61.61 g/L	Preferential exclusion
**Sugars—disaccharides**		
Sucrose	117.15 g/L	Preferential exclusion
Trehalose	117.15 g/L	Preferential exclusion
Lactose	117.15 g/L	Preferential exclusion
**Polyols**		
Glycerol	31.52 g/L	Preferential exclusion “solvophobic effect”
Mannitol	62.34 g/L	Preferential exclusion
Sorbitol	62.34 g/L	Preferential exclusion
**Methylamines**		
Betaine	40.09 g/L	Preferential exclusion
**Amino acids**		
Histidine	53.06 g/L	Preferential exclusion
Arginine*	59.58 g/L	Preferential exclusion
Methionine*	51.03 g/L	Preferential exclusion

*not used in this study

The cultures were incubated, induced and harvested according the results of the overexpression pretest. The periplasmic fraction was isolated as described above with the exception that insoluble proteins were removed by an additional centrifugation step. Subsequently, the soluble periplasmic fraction was analysed by SDS-PAGE and the soluble overexpressed protein was detected by Western Blot using antibodies that are specific for the fused affinity tag.

### Co-solvent assisted overexpression

For protein overproduction, the conditions that were determined to be optimal in the overexpression pretests and co-solvent screen were used in an up-scaled culture. A single colony of freshly transformed plates was picked to inoculate 30 ml of fresh LB-medium containing the desired antibiotic. The culture was incubated over night under shaking at the permissive temperature. A 4 liter main culture of LB medium supplemented with an iso-osmotic amount of the co-solvent was inoculated with 1% (v/v) of the preculture and substituted with the desired antibiotic. Cells were harvested by centrifugation and the pellets were frozen at -20°C.

### Co-solvent assisted purification under native conditions

Prior to lysis the cells were left thawing for 15 min on ice and resuspended in 8 ml buffer composed of 500 mM co-solvent, 1 mM EDTA and 100 mM Tris pH 8. The suspension was substituted with 1 mg/ml lysozyme and incubated for 30 min on ice. Subsequently benzonase was added to a final concentration of 20 U/ml and the suspension was incubated for additional 15 min on ice. The cleared lysate was prepared by centrifugation. For the cleared lysate obtained from 4 L culture 1 ml of resin was equilibrated in a PE column with 2 ml of buffer W (500 mM co-solvent, 1 mM EDTA, 100 mM Tris pH 8). The cleared lysate was allowed to enter the column by gravity flow. The column was washed 5 times with one column volume (CV) of buffer W. The protein was eluted 6 times with 0.5 CV of buffer E (500 mM co-solvent, 1 mM EDTA, 100 mM Tris, 150 mM NaCl, 2.5 mM desthiobiotin, pH 8) and eluates were collected separately.

### PBP6_Cp_ *in vitro* activity

Prior to the experiment, PBP6_Cp_ was exchanged into co-solvent free buffer (5 mM MES, pH 5.5) to avoid possible betaine-related effects on the thin layer chromatography (TLC) reaction product detection system (see below). Standard *in vitro* activity assay for PBP6_Cp_ was carried out in a final volume of 70 μl containing 0.1 nmol purified PBP6_Cp_, 2 nmol lipid II [13,14], 50 mM MES, pH 5.5, 2 mM MgCl_2_ and 20% DMSO. The reaction mixture was incubated for 4 h at 37°C.

### Detection and characterization of reaction products

Reaction products were extracted with 70 μl of n-butanol / pyridine acetate (2:1 v/v, pH 4.2) and the organic phase was analyzed by TLC and mass spectrometry (MS) as described before [15]. For TLC, silica was used as the stationary phase whereas the mobile phase consisted of chloroform-methanol-water-ammoniumhydroxide (88:48:10:1). Spots were visualized by phosphomolybdic acid (PMA) staining. For MS, the organic phase was dried under vacuum and acidified using TCA (20%). One microliter of this sample was placed onto a ground steel MALDI-TOF target plate and dried at room temperature. Each sample was overlaid with 1 μl of a saturated solution of 6-Aza-2-thiothymine in 50% ethanol/20mM diammonium citrate and dried at room temperature. The spectra were recorded in the reflector negative mode at a laser frequency of 9 Hz within a mass range from 300 to 3000 Da on a biflex III mass spectrometer (Bruker Daltonik GmbH, Bremen, Germany). Data were analyzed using flexAnalysis software (Bruker Daltonik GmbH). Instrument parameter settings: IS1 19 kV, IS2 16.5 kV, lens 9.5 kV, PIE 200 ns, Reflector 20kV, no gating.

## Results

### Co-solvents as *in vivo* stabilizing agents in periplasmic overexpression of recombinant proteins

We developed a lab-scale method for the periplasmic overexpression of proteins in *E*. *coli* that involves the systematic use of co-solvents *in vivo*. PBP6 from *C*. *pneumoniae* (PBP6_Cp_) was chosen as an ideal candidate for validating the co-solvent based system. *Chlamydiaceae* are evolutionary far away from the expression host *E*. *coli* and adopted to replicate in host derived inclusions whose physico-chemical properties are largely unknown [16]. Comparable to many other chlamydial proteins, PBP6_Cp_ is rich in cysteine (11 versus 1 cysteine residue in the *E*. *coli* homolog), a phenomenon that was discussed as adaptation to an unusal redox environment [17]. Consistently, all standard methods for heterologous overexpression in *E*. *coli*, including cytoplasmic overexpression, chaperone assisted overproduction, and periplasmic overexpression, failed to provide detectable amounts of the recombinant protein or resulted in insoluble protein aggregates (a complete list of unsuccessful approaches is provided in Table B in S1 File).

The workflow for our co-solvent assisted method is depicted in Fig 1a and comprises of (i) cloning into a periplasmic expression vector, (ii) small-scale pretests for overexpression, (iii) a small scale co-solvent screen, (iv) co-solvent assisted overexpression using the optimal combination of overexpression conditions and co-solvent as identified in the two steps before, and (v) purification of the protein in the presence of the co-solvent used before.

The PBP6_Cp_ encoding gene was cloned into an expression vector that allows for periplasmic expression and contains a tet promotor which is independent of the genetic background of the expression strain. The gene was N-terminally fused to the leader peptide OmpA which facilitates translocation and is cleaved afterwards by the *E*. *coli* host signal peptidase I. At the C-terminus, a tag for affinity chromatography-based purification was attached (Figs 1 and 2). Moreover, a putative C-terminal transmembrane domain in PBP6_Cp_ was removed to allow for accumulation of the protein in the soluble periplasmic fraction (Fig 2a).

In the next step, overexpression pretests were carried out to identify conditions that facilitate the production of PBP6_Cp_ and yield the highest amounts of (aggregated) protein. Variation of the *E*. *coli* expression strain (*E*. *coli* K-12 subsp. W3110, *E*. *coli* C43 (DE3), and *E*. *coli* JM83), the growth temperature (25°C and 30°C) and the time of induction (4h, over night) were used as parameters to affect overexpression. Under any conditions tested, PBP6_Cp_ was not produced in its soluble form. Overexpression in *E*. *coli* C43 (DE3), after 4h of induction at 25°C was identified to result in the highest amount of insoluble protein that was accumulated in the periplasmic pellet fraction (Fig 2b).

Following identification of optimal expression conditions, we developed a small-scale co-solvent screen to determine the optimal co-solvent for PBP6_Cp_. The screen includes 9 substances that are well established in stabilizing biologicals in biopharmaceutical formulation *in vitro* and listed in Table 1. Of note, osmotic stress was prevented by removing the standard amount of 10 g/L of sodium chloride (171 mM equivalent to 342 osmoles of dissociated Na^+^ and Cl^-^ ions per L (342 osm/L)) from the LB growth medium and replacing it with iso-osmotic concentrations of the respective non-dissociating co-solvent (342 osm/L equivalent to 342 mM). Toxic effects of amino acids were avoided by restricting concentrations to 50 mM and supplementing with 292 mM sucrose. Stress free conditions were further achieved by maintaining the growth temperature before and after induction at the same temperature. Our screen revealed that betaine assisted expression resulted in the highest yield of soluble PBP6_Cp_ in the periplasm (Fig 2c). To investigate whether our co-solvent platform is also applicable for other proteins we additionally screened three other chlamydial proteins (AmiA _Cp_, Cpn0902, GlyA_Cp_). For all proteins, the systematic screen of co-solvents helped to increase the yield of heterologous production (Figure A in S1 File).

The above determined parameters (*E*. *coli* strain C43 (DE3), 4h of induction at 25°C) were used in an up-scaled culture to overproduce soluble PBP6_Cp_ in the presence of betaine (Fig 2d). Based on the well established use of co-solvent as *in vitro* additives in biopharmaceutical formulation, purification of PBP6_Cp_ followed standard protocols for affinity tagged proteins with the exception that all buffers were supplemented with betaine to maintain stabilization of the protein over the whole process, making the protein assessable to biochemical characterization.

### Betaine assisted overexpression and purification of PBP6_Cp_ yields functional protein

**Fig 3:**
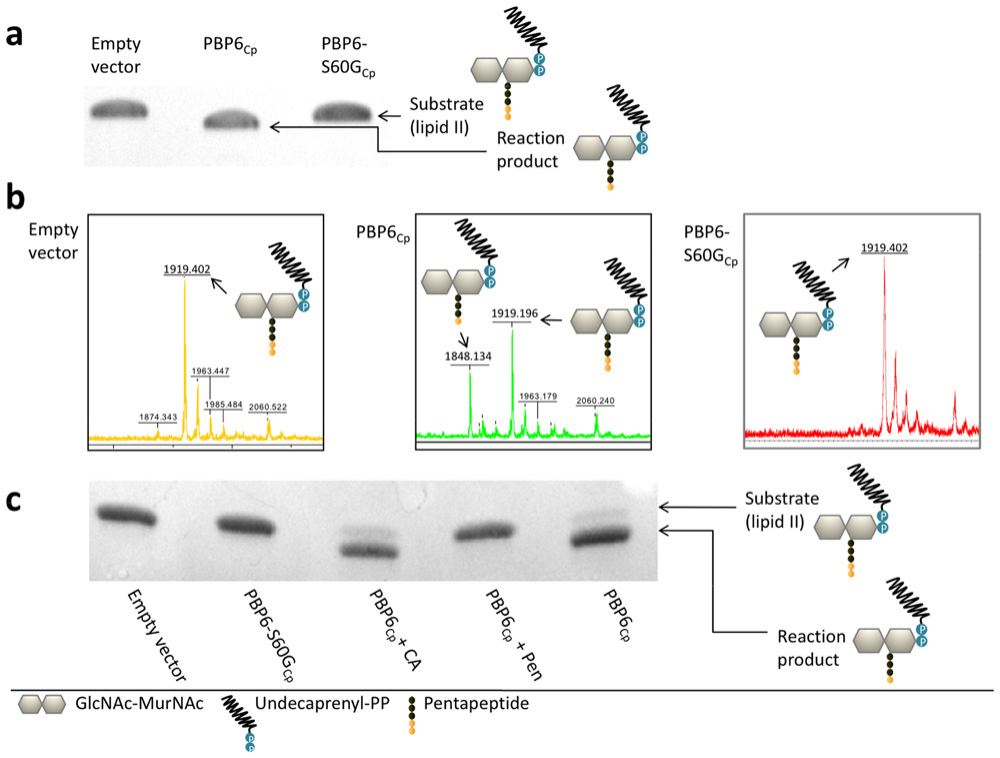
*In vitro* activity of PBP6_Cp_. The purified enzyme showed DD-carboxypeptidase activity on lipid II. (a, c) TLC and (b) MS analysis of reaction products. Cleaving of terminal D-Ala from the pentapeptide side chain of lipid II resulted in the formation of undecaprenyl-pyrophosphoryl-MurNAc-(GlcNAc)-tetrapeptide. (a,b) The exchange of S60 in the SxxK motif as well as (c) inhibition by penicillin G lead to a loss of function. CA: clavulanic acid; Pen: penicillin G.

*Chlamydiaceae* genomes encode only three PBPs [8]. Being homologs of PBP2 and PBP3 from *E*. *coli*, the two high-molecular-weight PBPs are predicted to serve as monofunctional transpeptidases which cross-link adjacent peptidoglycan strands in *E*. *coli* [8]. The only low-molecular-weight PBP is classified as a class C type 5 PBP having 29% amino acid sequence identity with *E*. *coli* PBP6. In conventional bacteria that are surrounded by a classical cell wall envelope, these enzymes function as DD-carboxypeptidases and modulate the mature peptidoglycan meshwork by cleaving terminal D-Ala from pentapeptide side chains that are not cross-linked [18]. After successful betaine-assisted overexpression and purification of soluble PBP6_Cp_, the protein was tested for *in vitro* activity. In the absence of a cell wall envelope in *Chlamydiaceae*, we tested the ability to cleave terminal D-Ala from the completed cell wall building block lipid II. Prior to the experiment, PBP6_Cp_ was rebuffered in co-solvent free buffer to avoid possible betaine-related effects on downstream reaction product detection. Thin layer chromatography (TLC) analysis as well as mass spectrometry revealed DD-carboxypeptidase activity on lipid II implicating that PBP6_Cp_ was overexpressed and purified in its active, correctly-folded form (Fig 3a and 3b).

### DD-carboxypeptidase active site mutagenesis and inhibition

To further investigate whether the observed degradation of lipid II is conferred by the catalytic activity of the purified PBP6_Cp_ protein we generated an active site mutant and analyzed inhibitory effects of penicillin. Active sites of PBPs are composed of three conserved motifs [18]. The SxxK motif contains the active site serine and is mainly involved in binding of the substrate forming a so called acyl-complex [19,20]. In case of the natural substrate, the D-Ala-D-Ala moiety of a pentapeptide side chain in peptidoglycan, the complex is transient whereas in case of beta-lactams, such as penicillin, the complex is long-lived and blocks enzymatic activity [19]. The two other motifs (S(Y)xN and K(H,R)T(S)G) exhibit coordinating functions and are responsible for substrate specificity. We found all motifs to be conserved in PBP6 homologs across *Chlamydiales* (Figure B in S1 File) and replaced S60 in PBP6_Cp_ (Fig 2a, corresponding to the serine residue in SxxK) via site directed mutagenesis. In contrast to the wild type the mutant protein was not capable of cleaving the terminal D-Ala from lipid II (Fig 2a and 2b). Moreover, DD-carboxypeptidase activity of wild type PBP6_Cp_ was stalled in the presence of penicillin G (Fig 2c). These results further proof that our method facilitated purification of PBP6_Cp_ in its active, correctly folded form and indicates that DD-carboxypeptidase activity of chlamydial PBP6 depends on a functional SxxK motif as typically shown for bacterial PBPs.

## Discussion

### Co-solvent assisted periplasmic expression and purification provides soluble and active recombinant protein

Since the launch of biologicals, such as peptide hormones, enzyme replacement therapeutics, and antibodies, new techniques were needed to achieve stability and long time storage of these products. As a consequence of this need co-solvents were intensively characterized and approved as additives in biopharmaceutical formulation [4]. These substances exhibit a high ability to preserve proteins in their native state [21]. We transferred the principle of co-solvent assisted *in vitro* stabilization of biologicals in industrial formulation to heterologous overproduction of proteins at the life science laboratory, where scientists are often confronted with denatured proteins which aggregate in inclusion bodies [2]. In 1990 Bowden and Georgiou added sugars to the growth medium during periplasmic expression of the TEM β-lactamase from *E*. *coli* and noticed a reduced aggregation of the protein [22]. Our approach was to test co-solvents that are well known in biopharmaceutical formulation systematically for *in vivo* stabilizing effects during overexpression of recombinant proteins in the periplasm of *E*. *coli*. The new method includes a small-scale co-solvent screen with the most common co-solvents in biopharmaceutical formulation (Fig 1a, Table 1). Subsequent overexpression as well as purification of the protein takes place in the presence of the co-solvent which was identified in the screen before to show best stabilization effects for the recombinant protein. In case of incompatibility with downstream applications the co-solvent can easily be removed by rebuffering the protein directly before use. With the potential to become a standard procedure, the rapid, cost-effective and easy handling lab-scale method yields in soluble and active recombinant protein. Moreover, the method might be particularly helpful for proteins that derive from evolutionary far afield organisms or originate from an unkown physico-chemical environment as demonstrated with the chlamydial protein PBP6 for which standard protocols failed to produce native protein. In line, our co-solvent platform proved to be beneficial for the heterologous production of three other chlamydial proteins (AmiA_Cp_, Cpn0902, GlyA_Cp_).

**Fig 4:**
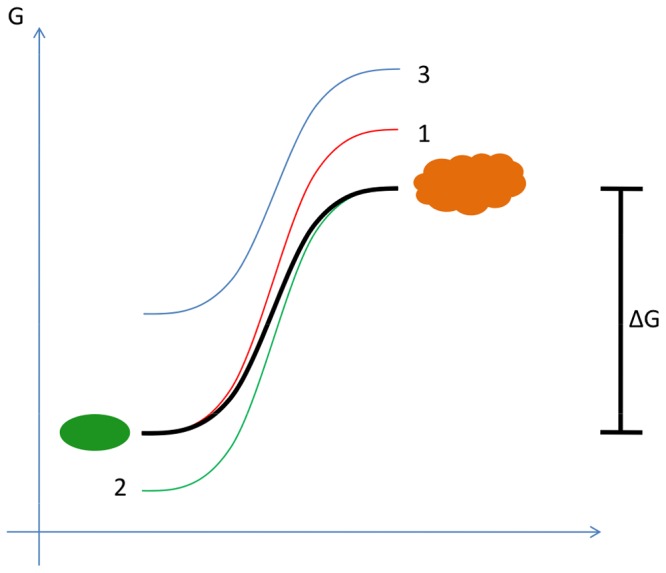
Energy levels of the denatured and native state of a protein. In the diagram ΔG is representing the free energy necessary to unfold the protein. In case 1, upon addition the co-solvent is excluded from the surface of the denatured state of the protein and by that increasing the energy level of the denatured state. In case 2, the co-solvent only binds to the native state of the protein and lowers the energy level. Case 3 illustrates the mode of action of most co-solvents to stabilize proteins. Exclusion of the co-solvent from both, the native and the denatured state, leads to an overall increased level of free energy. The green and the orange shape represent the protein in its native and denatured state, respectively.

To characterize the stabilizing effects of co-solvents on a thermodynamic level Wyman stated the following linked function: (δ ln K / δ ln a_x_)_T, P, a_ = ν_x Prod_—ν_x React_ = Δν_x_ (K = equilibrium constant, ν = binding of the ligand) [23]. It describes the influence of a substance (x) on either the native or the denatured state of a protein in dependency of the concentration (activity, a), temperature (T) and pressure (P). Based on the work of Wyman, Timasheff developed a model that describes the influence of co-solvents by showing the interactions between the co-solvent and the protein [5]. In general, denaturation of a protein requires a certain change in free energy (ΔG), whereby the cold denaturation represents a special case with a change in heat capacity [24]. To stabilize a protein you can lower the level of free energy of the native state (case 2), increase the level for the denatured state (case 1) or increase the overall energy level (case 3) (Fig 4). The latter case represents the underlying mechanism of “preferential exclusion” and the mode of action of most co-solvents including all substances used in our screen (Table 1).

The use of “preferential exclusion” based co-solvent stabilization of recombinant proteins during overexpression offers advantages over other methods. Chaperons are routinely used in lab scale to achieve soluble and active protein exploiting their ability to influence protein folding. Chaperones are more or less strongly bound to the target protein and coelution during purification can occur [25] making additional purification steps necessary. For our test protein, chlamydial PBP6, chaperone assisted expression trails failed to yield in soluble protein (Table B in S1 File). Another method mostly used in industry is refolding of pre-purified denatured protein aggregates. In lab scale the elaborative procedure is often infeasible due to the lack of high throughput screening systems for optimal refolding conditions. Moreover, the use of compatible-solutes under salt-induced osmotic stress conditions has been shown to allow for overproduction of functional immunotoxins in *E*. *coli* [26]. Compatible-solutes, also known as osmolytes are accumulated into the cytoplasm under osmotic stress conditions to counterbalance osmotic pressure. The underlying mechanisms of the hyperosmotic stress and compatible solute assisted immunotoxin expression is not completely understood [26]. In line with the observation that expression of soluble TEM β-lactamase in the presence of sugars, as described above, was not related to an increased osmotic pressure [22], the hyperosmotic stress assisted method did not succeed for our test protein (Table B in S1 File). In contrast, with our osmotic-stress free and co-solvent assisted system purification of a functional chlamydial PBP was achieved for the first time.

Our method could be adapted to other overexpression hosts like *Bacillus* or yeast in which proteins can be engineered for secretion to the outside of the cell and are directly accessible for stabilizing agents in the growth medium. Moreover, the co-solvents included in our screen meet all stipulated criteria from the pharmacopoeias and good manufacturing practice (GMP) guidelines like harmlessness and physico-chemical stability [4] and the method could be transferred to the production of food, drug and pharmaceutical products.

### Mechanistic insights in the chlamydial anomaly

In contrast to high-molecular weight PBPs the low-molecular weight PBPs from free living bacteria function as DD-carboxypeptidases or endopeptidases and lack the ability to incorporate lipid II into the peptidoglycan meshwork which maintains cell shape and resists osmotic challenges [18]. Cleaving terminal D-Ala from free pentapeptide side chains the DD-carboxypeptidases modulate the cell wall and are thought to play a role in maintenance of morphology and in septum formation [18,27]. In most conventional bacteria which are surrounded by a classical cell wall envelope several apparently dispensable and enzymatically redundant low-molecular weight PBPs are present, whereas cell-wall envelope lacking *Chlamydiaceae* possess only a single homolog. PBP6 from *E*. *coli* was discussed to be involved in stabilizing peptidoglycan in the stationary phase [18]. Moreover, *E*. *coli* PBP6 was implicated in the function of PBP3 by supplying substrate to the monofunctional transpeptidase during cell division [18]. Here we show that PBP6_Cp_ is functionally conserved in *Chlamydiaceae* and capable of removing D-Ala from the peptide side chain of cell wall building block lipid II *in vitro*. For the first time a chlamydial PBP was biochemically characterized and shown to be targeted by penicillin implicating that the enzyme contributes to penicillin induced persistence. Recently, cell wall sacculi could be detected in evolutionary older and genomically less reduced environmental chlamydiae [28]. However, a recent study provided evidence for the presence of distinct circular peptidoglycan-like structures in the “minimal bacteria” *Chlamydiaceae* suggested to be localized to the cell division site [29]. PBP6_Cp_ might not only use lipid II as a substrate but also modify this rudimentary ring-like structure *in vivo*. Recently, AmiA from *C*. *pneumoniae* was identified as a bifunctional enzyme with amidase as well as penicillin-sensitive DD-carboxypeptidase activity on lipid II [15]. Moreover, the LysM domain containing chlamydial protein Cpn0902 (NlpD) was shown to have DD-carboxypeptidase activity on lipid II [15], increasing the total number of DD-carboxypeptidases in *Chlamydiaceae* to three. Concerted action of PBP6, AmiA and Cpn0902 might confer DD-carboxypeptidase functions in chlamydial cell division. Moreover, as discussed for DD-carboxypeptidases from free-living bacteria [18], these enzymes might contribute to regulation of the release of immunogenic chlamydial muropeptides which are sensed by human cytosolic pattern recognition receptors Nod1 and Nod2 [8]. *In vitro* reconstitution of the penicillin sensitive catalytic activity of chlamydial PBP6 presented in this work opens up an avenue for further research on the physiological role of DD-carboxypeptidases in *Chlamydiaceae* which is needed to elucidate the still enigmatic chlamydial anomaly.

## Supporting Information

S1 File Supporting information. This file contains supporting tables (Table A in S1 File and Table B in S1 File) and figures (Figure A in S1 File and Figure B in S1 File).
(PDF)
