# Supplementary material for: Co-Solvents as Stabilizing Agents during Heterologous Overexpression in Escherichia coli – Application to Chlamydial Penicillin-Binding Protein 6
Source: PLoS One. 2015 Apr 7;10(4):e0122110. doi: 10.1371/journal.pone.0122110 (PMC4388811; doi:10.1371/journal.pone.0122110)
Supplement: S1 File — This file contains supporting tables (Table A in S1 File and Table B in S1 File) and figures (Figure A in S1 File and Figure B in S1 File). (PDF) [file pone.0122110.s001.pdf]

Supporting information

Table A in File S1. Strains, plasmids and primers used in this study.

Strain/ primer	Application/ description
E. coli TOP10	Cloning strain, Invitrogen (Life Technologies, Darmstadt, Germany)
E. coli W3110	Overexpression strain, wild type strain, DSMZ (DSM5911)
E. coli JM83	Overexpression strain, recommended for periplasmic overexpression [1], DSMZ (DSM3947)
E. coli C43 (DE3)	Overexpression strain; Lon and OmpT protease deficient, Lucigen (Middleton, WI)
E. coli HM125	Overexpression strain; DegP protease deficient [3]
PBP6ΔSPΔTM_for_Cp	ATGGTAGGTCTCAGGCCGGACTCTCTTTCCCAGAAGTACG
PBP6ΔSPΔTM_rev_Cp	ATGGTAGGTCTCAGCGCTGACACGTTTCATATAGAGTTTCCAA
PBP6ΔSPΔTM_S60G_for_Cp	CTGTAATCTATCCTGCCGGCATGACGAAAATCGCA
PBP6ΔSPΔTM_S60G_rev_Cp	TGCGATTTTCGTCATGCCGGCAGGATAGATTACAG
pASK-IBA2C_PBP6Cp	*dacF* coding for PBP6 from *C. pneumoniae,* the intrinsic N-terminal signal peptide is replaced by the OmpA leader peptide, C-terminal Strep-tag, plasmid used for periplasmic overproduction
pASK-IBA2C_PBP6S60GCp	*dacF* mutant coding for PBP6 S60G variant from *C. pneumoniae,* the intrinsic N-terminal signal peptide is replaced by the OmpA leader peptide, C-terminal Strep-tag, plasmid used for periplasmic overproduction and active site studies

Table B in File S1. Methods that failed to produce soluble $PBP6_{Cp}$.

location	leader peptide	affinity tag	promotor	vector	modification of $PBP6_{Cp}$	*E. coli* expression strain	growth temperature	time of induction	remark	result
CP	-	His, C-t	T7	pET21b	-	BL21	RT	3h, o/n	deficient in Lon and OmpT proteases	-/-
CP	-	His, C-t	T7	pET21b	-	BL21	RT	3h, o/n	v.s., chaperones included (Dnak, DnaJ, GrpE)	insoluble protein
CP	-	His, C-t	T7	pET21b	-	BL21	RT	3h, o/n	v.s., chaperones included (GroES, GroEL)	insoluble protein
CP	-	His, C-t	T7	pET21b	-	C43(DE3)	RT	3h, o/n	optimized for expression of membrane proteins	-/-
CP	-	Strep, C-t	tet	pASK-IBA3	-	BL21	RT	3h, o/n	deficient in Lon and OmpT proteases	-/-
CP	-	Strep, C-t	tet	pASK-IBA3	-	Origami™ (DE3)	25 °C, 30 °C	4h, o/n	enhanced disulfide bond formation	-/-
CP	-	Strep, C-t	tet	pASK-IBA3	ΔSP	Origami™ (DE3)	25 °C, 30 °C	4h, o/n	v.s.	-/-
CP	-	Strep, C-t	tet	pASK-IBA3	ΔTM	Origami™ (DE3)	25 °C, 30 °C	4h, o/n	v.s.	-/-
CP	-	Strep, C-t	tet	pASK-IBA3	ΔSPΔTM	Origami™ (DE3)	25 °C, 30 °C	4h, o/n	v.s.	-/-
PP	OmpA	Strep, C-t	tet	pASK-IBA2	-	JM83	25 °C, 30 °C	4h, o/n	recommended for periplasmic overexpression [1]	-/-
PP	OmpA	Strep, C-t	tet	pASK-IBA2	ΔSP	JM83	25 °C, 30 °C	4h, o/n	v.s.	-/-
PP	OmpA	Strep, C-t	tet	pASK-IBA2	ΔTM	JM83	25 °C, 30 °C	4h, o/n	v.s.	-/-
PP	OmpA	Strep, C-t	tet	pASK-IBA2	ΔTM	JM83	25 °C, 30 °C	4h, o/n	v.s., method from Barth et al. [2]	insoluble protein
PP	OmpA	Strep, C-t	tet	pASK-IBA2	ΔSPΔTM	JM83	25 °C, 30 °C	4h, o/n	v.s., method from Barth et al. [2]	insoluble protein
PP	OmpA	Strep, C-t	tet	pASK-IBA2	ΔSPΔTM	JM83	25 °C, 30 °C	4h, o/n	Recommended for periplasmic overexpression [1]	insoluble protein
PP	OmpA	Strep, C-t	tet	pASK-IBA2	-	C43(DE3)	25 °C, 30 °C	4h, o/n	Lon and OmpT deficient, membrane and toxic proteins	-/-
PP	OmpA	Strep, C-t	tet	pASK-IBA2	ΔSP	C43(DE3)	25 °C, 30 °C	4h, o/n	v.s.	-/-
PP	OmpA	Strep, C-t	tet	pASK-IBA2	ΔTM	C43(DE3)	25 °C, 30 °C	4h, o/n	v.s.	insoluble protein
PP	OmpA	Strep, C-t	tet	pASK-IBA2	ΔSPΔTM	C43(DE3)	25 °C, 30 °C	4h, o/n	v.s.	insoluble protein
PP	OmpA	Strep, C-t	tet	pASK-IBA2	-	HM125	25 °C, 30 °C	4h, o/n	DegP protease deficient [3]	-/-
PP	OmpA	Strep, C-t	tet	pASK-IBA2	ΔSP	HM125	25 °C, 30 °C	4h, o/n	v.s.	-/-
PP	OmpA	Strep, C-t	tet	pASK-IBA2	ΔTM	HM125	25 °C, 30 °C	4h, o/n	v.s.	-/-
PP	OmpA	Strep, C-t	tet	pASK-IBA2	ΔSPΔTM	HM125	25 °C, 30 °C	4, o/n	v.s.	insoluble protein
PP	OmpA	Strep, C-t	tet	pASK-IBA2	ΔTM	W3110	25 °C, 30 °C	4h, o/n	Wild type	-/-
PP	OmpA	Strep, C-t	tet	pASK-IBA2	ΔSPΔTM	W3110	25 °C, 30 °C	4h, o/n	Wild type	-/-

CP: cytoplasma, PP: periplasma, SP: signal peptide, TM: transmembrane domain, -/-: overproduced protein was not detectable, v.s.: see above, o/n: overnight, N-t: N-terminal, C-t: C-terminal.

Figure A in File S1. Co-solvent screen for AmiA$_{Cp}$, CPn0902, and GlyA$_{Cp}$.

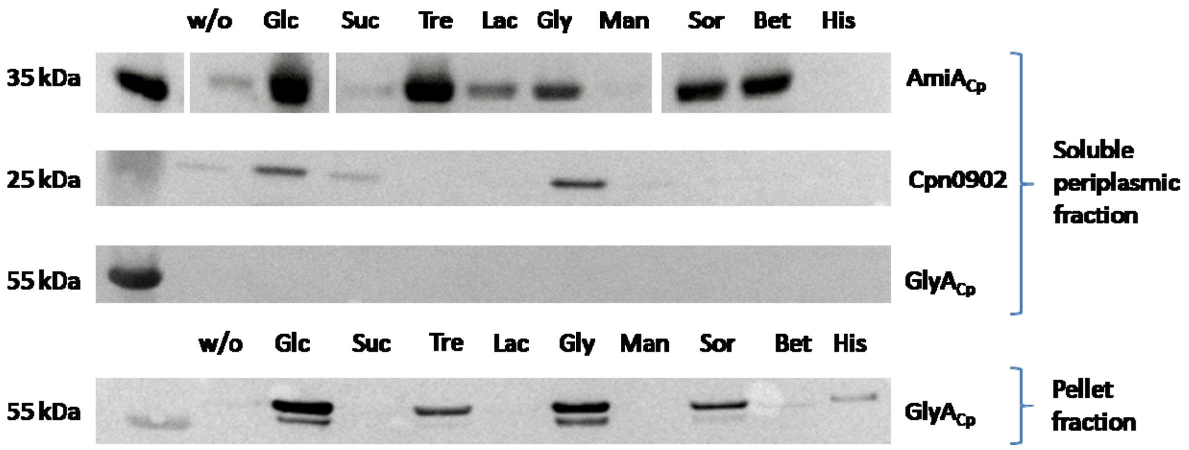

Heterologous production of chlamydial proteins AmiA$_{Cp}$, Cpn0902 and GlyA$_{Cp}$ benefits from the co-solvent platform. Addition of co-solvents increases the yield of soluble AmiA$_{Cp}$ and Cpn0902 in the periplasm to varying extent. In the case of the co-factor PLP dependent enzyme GlyA$_{Cp}$ the addition of co-solvents does not rescue the protein from the pellet fraction but increases the amount of insoluble protein. w/o: without the addition of co-solvent, Glc: glucose, Suc: sucrose, Tre: trehalose, Lac: lactose, Gly: glycerol, Man: mannitol, Sor: sorbitol, Bet: betaine, His: histidine.

Figure B in File S1. Sequence alignment of *E. coli* PBP6 with *Chlamydiaceae* and environmental chlamydiae species.

```
C.pneumoniae        ------------------------MKRPFFTYLCIIFYGS--CASLSLHAGLSFPEVRGA 34
C.trachomatis       -------------------------MRTFFLLCRFFICLAPFFLSFPLYADPHTVLTKGI 35
C.psittaci          ------------------------MIRVFFRFALLPFLVG--LFTHPLYGHIVFPETRGN 34
P.acanthamoebae     -----------------------------MLKLTYYLIISLLFFSNSLFSNTLDVFVEAE 31
Pr.amoebophila      MIFNFLVVFFYNFSDFIRNILVSKRQVEHGFKMRVWYIFFVTIISFSLRSAPLDFKIAGE 60
W.chondrophila      ------------------------------MNSTFCLIFALLLANSILNG--IEIKIHAD 28
S.negevensis        ------------------------------MFYVRFWILFLVCLCQYTWAEP-LKVKVTAK 30
E.coli              --------------------MTQYSSLLRGLAAGSAFLFLFAPTAFAAEQTVEAPSVDAR 40
                                                                              .

C.pneumoniae        TAAVVHADSGKVFYDKDIDAVIYPASMTKIATALFI---LKHYPTVLDTLIKVKQDAIAS 91
C.trachomatis       AAAVVHADSGAILKEKNLDHKIFPASMTKIATALLI---LRQYPDVLTRFITTRREPLTS 92
C.psittaci          AVAVVHAETGKVLYAKDIDKRIYPASMTKIATALFI---LKKHPDVLNRFIIVKPDAIAS 91
P.acanthamoebae     AAILMNAETGKILYEKNIHTSYFPASITKIATAAFV---LKFFSDKLDVIVTAEQDSIGW 88
Pr.amoebophila      AAILLNAESGAILFEQEAYSCQFPASTTKVATALYA---LSKKQD-LNQMMAAEQDSLTT 116
W.chondrophila      SAILMNADTGAILYEKNGKKEHFPASLTKIATAIYT---LQLREQKLDKMLTAEHEAIAS 85
S.negevensis        SAILINAKTGAILYEKAPHDPHYPGSITKVATLTYA---LSQYNESYDEVVTCPYHCLKK 87
E.coli              AWILMDYASGKVLAEGNADEKLDPASLTKIMTSYVVGQALKADKIKLTDMVTVGKDAWAT 100
                    :  ::.  :* ::          *.* **: *       *         .:    .

C.pneumoniae        ITPQAKKQSGYRSPPHWLETDGSTIQLHLREELLGWDLFHALLVCSANDAANVLAMACCG 151
C.trachomatis       ITPQAKQQSGYRSPPHWLETDGMTIQLKVKEEVSGWDLFHALLISSANDAANVLADACCQ 152
C.psittaci          ITPQAKKQSGYRSPPHWLETDGVTIQLQNKEEVSGWDLFHALLICSANDAANALAIACSG 151
P.acanthamoebae     VTEEAKRKSNYTLPAYWLVPGGTHMGIMKGEQLSLEDLLHGLMLVSANDSANVIAQYVGG 148
Pr.amoebophila      LSQEAKKKTNYDPP-YRLEPDGSHIGLKKGEMMSLHDLLGGMLIPSGNDAANVIANALGP 175
W.chondrophila      VSEEEMVRSNYSLPAYWLTRGTSHIGIKKGEELSLRDLLYGMMVASGGDASNMIALYMGG 145
S.negevensis        INGSMKEAHLYRDPAYWLEPDGTHFWIKRGERLSMRDLLYGMMLVSGNDASNFIAHHVGG 147
E.coli              GNPALR--------------GSSVMFLKPGDQVSVADLNKGVIIQSGNDACIALADYVAG 146
                     .                  .   : :   : :   **  .::: *..*:.  :*

C.pneumoniae        SVEKFMDKLNFFLKEEIGCTHTHFNNPHGLHHPNHYTTTRDLISIMRCALK-EPPFRGVI 210
C.trachomatis       SVSAFMRQLNEFLR-ELGCQNTHFNSPHGLHHPDHYTTARDLSLIMKEALK-EPLFRQVI 210
C.psittaci          SVVEFMKQLNDFLR-EIGCAHTHFNNPHGLHHPNHYTTAGDLTRIMREGLK-EPLFRQVI 209
P.acanthamoebae     TVPDFVDQLNAYAK-QAGCQHTTFHNPHGLHHPEHKTTAYDMALLAKEALS-IPAFCKMF 206
Pr.amoebophila      TIPVFMAELNIYLK-KIGCEKTTFYNPHGLHHPQHQTTAYDLALITKEALK-QPVFCELI 233
W.chondrophila      TIPVFMEELNLYLK-ELGCTSTYLMNPHGLHHPRHVSTAYDMAVLTREALK-NSTFREIV 203
S.negevensis        TIPKFMRGLNDYIK-KLGCKNTYFANPHGLHHPKHVTSAYDMALIMQDAIK-NKIALEIL 205
E.coli              SQESFIGLMNGYAK-KLGLTNTTFQTVHGLDAPGQFSTARDMALLGKALIHDVPEEYAIH 205
                    :   *:  :* : : *   * : . ***. * : ::: *:  : :  :        :

C.pneumoniae        STTSYKIGATNLHGERILSPTNKLLLPGSTYHYPPALGGKTGTTKTAGKNLIMAAEKNNR 270
C.trachomatis       HTASYTMEATNLSPERVLSSTNKLLSSSSTYFYPPCLGGKTGTTKSAGKNIIFAAEKNNR 270
C.psittaci          RTTNYKMAPTNLSQERILNLTNKLILPGSTYYYPPALGGKTGTTKDAGKNLVFAAKKHGR 269
P.acanthamoebae     GTVKYTRPKTNKQESSVLVQNHPLLKQG-KFYYAKALGAKTGYTNLAAHNLVVAARDKDR 265
Pr.amoebophila      GQKKYLRPKTNKQAATTLLQTNRLIRPG-SFYYSKAIGGKTGYHAKAKKTYIGVARSEER 292
W.chondrophila      KTVRCQRPETNKQKATTLIQTNRLLRKG-KYYYSKAIGVKTGYTSQAKNNLVAAAKDGDR 262
S.negevensis        ATKEYERSATNMQSARIIQNTGLLLQPG-KFFYPKVIGMKNGYHSHAKHTFVGAAQQGDR 264
E.coli              KEKEFTFNKIRQPNRNRLLWSSNLNVDG----------MKTGTTAGAGYNLVASATQGDM 255
                              .      :  .  *   .           *.*    *  . :  * .

C.pneumoniae        LLVTIATGYSGPVSDLYQDVIALCETVFNEPLLRKELVP--PSDCLQLEIANLGKLSCPL 328
C.trachomatis       SIIVVAAGYFGPAAQLYQDAIALCEDLFNEQLLRCFLIP--PASHYPVPTR-FGTVTAPV 327
C.psittaci          SIITIATGYS-VMSELYEDVIALCEGVFNEQPLRRYLIP--PTEKYTLRLGLLGKISIPL 326
P.acanthamoebae     TLIAVLLKTK-ERDQLFKDAIKLFETAFQQPKVERLLLRQGIQKFTQKPNWASQPLKAYL 324
Pr.amoebophila      TLIVVLLGYQ-ERNTIFQDAIKLFDAAFNQPKIQRTFLKEGRQSFAHNLPKAACPLQTHL 351
W.chondrophila      TLIAVFMHCD-DREKMFLDAKQLFNKAFKEEKISRKVFQAGPQKMTLRVEGAAKSIATYI 321
S.negevensis        ILIAVLLECE-DPKQKYRDAIRLFEAAFSQEEETRLLFNKDENYFTREIKKGKTPLRASL 323
E.coli              RLISVVLGAK-TDRIRFNESEKLLTWGFRFFETVTPIKPDATFVTQRVWFGDKSEVNLGA 314
                     :: :           : :   *    *        .                  :

C.pneumoniae        PEGLYYDFYASED-REPLSVSFIAHADAFPIEQGDLLGHWVFYDDEGKKISSQPFYAPCR 387
C.trachomatis       AQGIYYDFYPSEG-DPLLTLSLEPNKISFPIRKGDLLGHWILSTPTGENQHSIPFLAESD 386
C.psittaci          PHGVYYDFYASEG-EEPKTLSFVPHATKLPIHKGDLLGHWVFRNISGERVRAEPLYAADA 385
P.acanthamoebae     DEDVKISYFSAE--EPQVQCAVEWLNLEPPIQKGQTIGQVCLTDREGAFKKYVPVKAYEE 382
Pr.amoebophila      KEPLKLDYYLAE--DPEAKCLLYWMIPPLPIQKDQQVGELHLIAKNGSLIKKASLLALGD 409
W.chondrophila      KNDVVFDFYPSE--EPKLKCLLKWNAVSLPVQKDQQVGVLVFEDDNG--KELHSEALFAK 377
S.negevensis        MENVLINYFPSE--EPEISIELNWEQLELPIAAGEWVGEMHILDQNHKILEKAPLYATRD 381
E.coli              GEAGSVTIPRGQLKNLKASYTLTEPQLTAPLKKGQVVGTIDF-QLNGKSIEQRPLIVMEN 373
                     .        .:         .       *:  .: :*   :           .

C.pneumoniae        FERTI-KPWKLYMKRVFTSYRTYMSITMLLMYFRIRKHRKYKNLKHYSKI-- 436
C.trachomatis       ILPTLKQRILLMSLRMLTTYRTYVLILLFVWIYRRKKQPRATKTFSNPFFS- 437
C.psittaci          IHPSIGQKIRLYTKRVMTSYRTYIVLTLVLLYYRKTRVHRRKSSRYYL---- 433
P.acanthamoebae     LSASWSFWIKQSFGELLFWHKATKIFAIVCILLFFG-GLVFQFRRR------ 427
Pr.amoebophila      VSYAWPYSWMANIEGFFG--QLSWLWVSLISLFMFG-LLFFIWKTSPKEL-- 456
W.chondrophila      EKVDHHFFRR--LRDRITGRKILKFFGACLAVLFIG-GLIYELGCVKKSSSG 426
S.negevensis        VKKKSFIAFVDGLKTGSLDFTPLRNVLIVFLLLAVGSTLYFVFKSNRSVK-- 431
E.coli              VEEGGFFGRVWDFVMMKFHQWFGSWFS------------------------- 400
```

The PBP active site motifs SxxK, S(Y)xN and K(H,R)T(S)G are boxed. *Chlamydia pneumoniae* (AAD18811.1), *Chlamydia trachomatis* (WP_009872745.1) *Chlamydia psittaci* (WP_015385937.1), *Parachlamydia acanthamoebae* (YP_004653031.1), *Protochlamydia amoebophila* (YP_007393.1) *Waddlia chondrophila* (YP_003709874.1), *Simkania negevensis* (WP_013944493.1), *Escherichia coli* (NP_415360.1).

References

1. Terpe K. Overview of bacterial expression systems for heterologous protein production: from molecular and biochemical fundamentals to commercial systems. Appl Microbiol Biotechnol. 2006;72: 211-222.

2. Barth S, Huhn M, Matthey B, Klimka A, Galinski EA, Engert A. Compatible-solute-supported periplasmic expression of functional recombinant proteins under stress conditions. Appl Environ Microbiol. 2000; 66: 1572-1579.

3. Meerman HJ, Georgiou G. Construction and characterization of a set of *E. coli* strains deficient in all known loci affecting the proteolytic stability of secreted recombinant proteins. Biotechnol. 1994;12: 1107-1110.
